# RFD-BiSeNet V2: A Lightweight Floodwater Segmentation Network for Vision-Based Environmental Sensing

**DOI:** 10.3390/s26092841

**Published:** 2026-05-01

**Authors:** Xinyan Li, Yining Shi, Sijie Wang, Jinghui Xu

**Affiliations:** College of Water Resources and Architectural Engineering, Northwest A&F University, Yangling, Xianyang 712100, China; yanzii_lee@nwafu.edu.cn (X.L.); shiyiningg@163.com (Y.S.); 15877514586@nwafu.edu.cn (S.W.)

**Keywords:** flood disaster, BiSeNet V2, lightweight semantic segmentation

## Abstract

**Highlights:**

**What are the main findings?**

- A lightweight RFD-BiSeNetV2 network is developed for accurate floodwater segmentation.
- The proposed model achieves a high mean Intersection over Union (mIoU) of 97.10% while enabling real-time inference.

**What are the implications of the main findings?**

- The model can be effectively deployed in real-time flood monitoring systems.
- The framework provides a practical solution for rapid flood detection and environmental disaster management.

**Abstract:**

Flood disasters pose significant threats to human life and infrastructure, creating an urgent need for reliable vision-based environmental sensing technologies for rapid floodwater identification. Vision-based platforms such as unmanned surface vehicles (USVs) provide an effective solution for monitoring inland water environments; however, accurate floodwater segmentation remains challenging due to complex water boundaries, reflections, and background interference. To address these issues, we propose RFD-BiSeNet V2, a lightweight semantic segmentation network. Building upon BiSeNet V2, our model integrates an edge-aware learning strategy to track dynamic contours, a feature refinement module to suppress reflection noise, and a multi-scale feature fusion module to accommodate varying morphological scales. Evaluated on a comprehensive dataset incorporating USV data, UAV imagery, and diverse real-world scenes, RFD-BiSeNet V2 achieves an mIoU of 97.10%, outperforming the baseline by 6.68%. Crucially, the results demonstrate the practical implications of our architectural advancements: the edge-aware and feature refinement modules successfully sharpen ambiguous water boundaries and effectively filter out severe surface reflections, directly driving the segmentation accuracy. With a compact size of 5.95M parameters and real-time inference capabilities, the model offers a robust and highly efficient solution suitable for resource-constrained deployments across diverse intelligent environmental sensing systems.

## 1. Introduction

Floods are among the most destructive natural disasters, posing serious threats to human life, property, and critical infrastructure. In recent years, enhancing urban resilience and developing comprehensive socio-physical mitigation strategies through advanced hydrological and infrastructure modeling have become critical focuses in broad disaster management [1,2,3]. Within this broader context of flood hazard mitigation, the rapid and accurate visual monitoring of inundated areas is an essential prerequisite for immediate emergency response. With the rapid development of intelligent environmental monitoring technologies, vision-based sensing platforms such as unmanned surface vehicles (USVs) and unmanned aerial vehicles (UAVs) have become increasingly important for observing inland water environments and detecting floodwater areas. However, accurate floodwater segmentation remains challenging due to the dynamic morphology of water boundaries, complex environmental backgrounds, and strong reflections on the water surface. Addressing these challenges during emergency response requires real-time, on-device processing capabilities; therefore, developing a lightweight model that avoids the high computational overhead of traditional deep networks while maintaining strong anti-interference ability is essential. Therefore, developing efficient and robust semantic segmentation algorithms for floodwater detection has become an important research topic.

### 1.1. Current State of Research

Traditional image processing methods primarily segment water bodies based on visual features such as color, texture, reflective characteristics, and grayscale values. Edge detection techniques like the Sobel and Canny operators are widely used for water boundary extraction [4,5]. Kröhnert et al. (2016) proposed a water shoreline detection method based on spatiotemporal grayscale value changes, but it is prone to detection deviations in complex water boundary scenarios [6]. Sun et al. (2019) employed superpixel segmentation technology for land–sea demarcation in satellite images [7]. Yu et al. (2020) extracted pixel texture and color features using Local Binary Patterns and grayscale covariance matrices, achieving water detection by incorporating the orientation and distance relationships of adjacent pixels [8]. While these methods have their respective merits, they often suffer from insufficient segmentation accuracy and low processing efficiency in complex water-land boundary scenarios and under specific lighting conditions.

With the advancement of artificial intelligence, deep learning-based water segmentation methods have gradually become mainstream. The proposal of the Fully Convolutional Network (FCN) broke through the limitations on input image size, laying a theoretical foundation for water segmentation network architecture design [9]. Classical semantic segmentation networks such as DeepLab, SegNet, and UNet further improved segmentation performance by introducing mechanisms like dilated convolution and skip connections [10,11]. Compared to traditional image processing methods, deep learning approaches hold significant advantages in feature learning capability and adaptability to complex scenes. Akiyama et al. (2020) processed images captured by sensors using a SegNet-based network [12]. Ling et al. (2022) introduced a differentiable binarization method based on the UNet architecture, effectively enhancing water segmentation accuracy while maintaining real-time performance [13]. Deep learning methods demonstrate stronger robustness against interfering factors such as water reflections, dynamic morphological changes, and fluctuating illumination, effectively avoiding the over-segmentation or under-segmentation problems common in traditional methods. Zhan et al. (2020) and Bovcon et al. (2021) showed that multi-level feature fusion mechanisms in deep learning facilitate the effective combination of low-level detail features and high-level semantic features, allowing for precise modeling of gradual characteristics in weak boundary regions [14,15]. Building on these mechanisms, recent structural innovations continue to improve feature extraction in complex disaster scenarios. For instance, Sener et al. (2024) designed a novel convolutional neural network incorporating a hybrid attentional atrous convolution module, which significantly enhances the precise detection of flood-affected areas by expanding the receptive field while maintaining attention on critical edge features [16]. Gebrehiwot et al. (2019) demonstrated the practicality and high accuracy of deep learning in disaster emergency remote sensing [17]. The development of this field has been further catalyzed by the emergence of standardized high-resolution benchmarks. Specifically, the version of the FloodNet dataset presented by Rahnemoonfar et al. (2021) has established a critical platform for post-flood scene understanding, enabling the evaluation of segmentation models on complex urban flood imagery captured from a UAV perspective [18].

In practical applications, water segmentation tasks require not only boundary precision but also must meet the demands of inference speed and real-time performance [19]. Existing research primarily enhances speed by reducing input size or simplifying network structures, but this often leads to loss of detail or diminished feature representation capability, making it difficult to balance accuracy and efficiency [20,21]. Addressing this trade-off, BiSeNet innovatively employed a dual-branch model structure, providing a feasible solution for balancing accuracy and real-time performance [22]. Building upon BiSeNet, Yu et al. (2021) optimized the branch structure and introduced an aggregation layer as a feature fusion module, proposing the BiSeNet V2 model, which further enhanced model performance [23].

Beyond algorithmic constraints, practical flood monitoring introduces physical acquisition complexities. Data is predominantly captured via mobile platforms (e.g., USVs and UAVs), whose continuous motion makes ascertaining the true camera pose relative to the scene highly difficult. To process imagery from these agile platforms, researchers have recently explored diverse algorithmic paradigms. For UAV-specific applications, Simantiris and Panagiotakis (2024) demonstrated the viability of an unsupervised color-based methodology for flood segmentation, while Shi et al. (2024) introduced a cutting-edge interactive prompt-based network that leverages visual foundation models to extract urban flood areas [24,25]. Compounding this issue, flood environments—particularly water surfaces and inundated shorelines—exhibit repetitive, low-texture patterns with a critical scarcity of distinct keypoints. Together, these unstable viewpoints and feature-sparse conditions largely preclude conventional geometry-based pipelines, such as 3D reconstruction and Structure from Motion (SfM). Consequently, 2D data-driven semantic segmentation emerges as a highly robust alternative, as it fundamentally bypasses the reliance on precise geometric constraints and stable feature matching.

### 1.2. Contributions and Novelty of This Survey

Although deep learning methods have achieved remarkable progress in water segmentation tasks, many existing models require substantial computational resources and contain large numbers of parameters, which limits their deployment in resource-constrained sensing platforms. To address these challenges, this study focuses on lightweight model design and proposes a flood segmentation algorithm named RFD-BiSeNet V2 (Refined Flood Detection BiSeNet V2). The proposed method aims to balance segmentation accuracy and computational efficiency for real-time flood monitoring applications.

The main contributions of this paper are summarized as follows:

- A lightweight floodwater segmentation network based on BiSeNet v2 is proposed to improve real-time performance in vision-based environmental sensing systems.
- An edge-aware learning strategy and feature refinement mechanism are introduced to enhance boundary perception and segmentation accuracy in complex flood scenes.
- A multi-scale feature fusion design is developed to improve feature representation capability while maintaining model efficiency.

## 2. Materials and Methods

### 2.1. Flood Dataset

**Figure 1:**
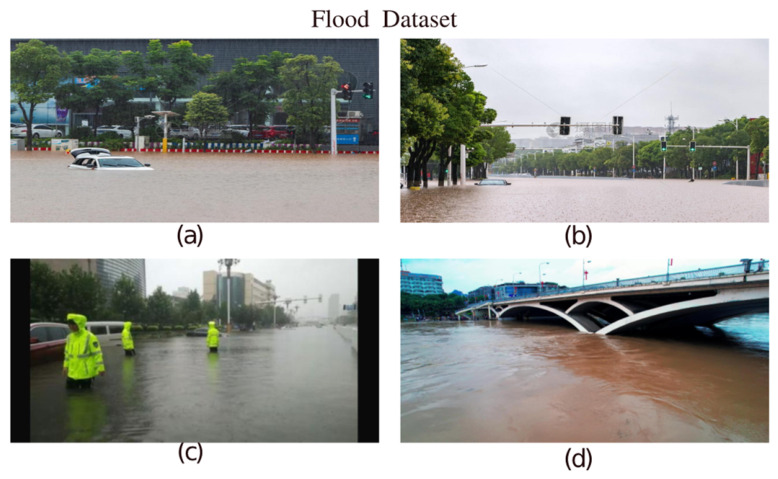
Visualization of the flood dataset (Figures (**a**–**d**) show a selection from the dataset).

To construct a robust and highly diverse flood inundation dataset (illustrated in Figure 1), this study integrated images from three distinct sources: the inland waterway dataset USVInland [26], the high-resolution flood dataset GF-FloodNet [27], and a custom collection of flood images collected from publicly available online resources. Geographically, all images within these integrated datasets were specifically sourced from various flood-affected regions across China. Compared to relying on a single source, this composite dataset encompasses a much broader array of flood scenarios, featuring varying water levels, complex topographic backgrounds, and diverse illumination conditions across multiple time periods. All water bodies within the dataset were meticulously annotated to ensure that the water contours align precisely with reality, providing high-quality ground truth samples. To standardize the input for model training, all images were uniformly resized to 640 × 320 pixels.

Initially, a total of 1291 strictly independent water area images were curated. To ensure rigorous model evaluation and prevent any potential data leakage, these original images were randomly partitioned into a training set and a validation set at an 8:2 ratio prior to any data augmentation. This strict scene-level separation guarantees that the validation set remains entirely unseen by the model during training, thereby substantiating the reliability of the evaluation metrics. Subsequently, to enhance the model’s generalization capability and prevent overfitting, the original training set underwent extensive data augmentation. Spatial-geometric transformations were applied independently to accommodate the varying spatial distributions of floodwaters. Furthermore, pixel-level augmentations, including brightness adjustments and noise injection, were employed to simulate real-world environmental interference. Through these targeted augmentations, the training set was expanded to 10,730 samples, providing a comprehensive foundation for robust model optimization.

### 2.2. Model Overview of BiSeNet V2

**Figure 2:**
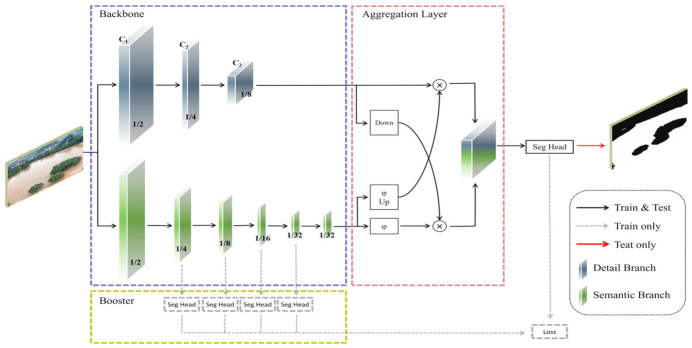
BiSeNet V2 network architecture diagram.

To address the inherent trade-offs between spatial details and contextual semantics—and consequently, between accuracy and real-time speed—BiSeNet introduced a highly efficient dual-path architecture [22,25]. Specifically, the Spatial Path employs a shallow network with limited downsampling to preserve high-resolution features and precise boundary details. Concurrently, the Context Path leverages deep architectures and attention mechanisms to capture global semantic representations. Building upon this paradigm, BiSeNet V2 further optimizes the dual-branch structure, serving as the foundational baseline for our proposed method (illustrated in Figure 2).

BiSeNet V2 typically employs cross-entropy as its core loss function:

(1) $$L_{main}=-\frac{1}{N}\sum_{i=1}^{N}\sum_{c=1}^{C}y_{i,c}\log(p_{i,c}),$$

where N represents the pixel class. C is the number of classes. $y_{i,c}$ is an indicator function determining whether the pixel belongs to class c. $p_{i,c}$ is the predicted probability after applying the softmax function to the network output.

For the flood segmentation task, this can be simplified as:

(2) $$L_{main}=-\frac{1}{N}\sum_{i=1}^{N}[y_{i}\log(p_{i})+(1-y_{i})\log(1-p_{i})],$$

Despite the dual-path architecture of BiSeNet V2, our baseline experiments reveal three critical limitations when applied to complex floodwater segmentation. First, while the spatial path is designed to capture low-level details, the model’s aggregation mechanism overwhelmingly prioritizes global semantics. Consequently, local spatial features are suppressed rather than preserved, causing edge information to severely attenuate and resulting in blurred water-land boundaries.

Second, flood boundaries inherently exhibit highly dynamic, multi-scale morphological variations. The baseline’s reliance on single-scale feature processing fails to capture these diverse edge structures, fundamentally restricting high-precision delineation.

Finally, floodwaters frequently present heterogeneous, turbid visual profiles due to suspended sediment and debris. These characteristics closely mimic adjacent non-flood backgrounds, such as damp roads or waterlogged vegetation. Lacking a dedicated feature refinement mechanism to filter this interference, BiSeNet V2 struggles to isolate discriminative features, leading to severe misclassification in easily confused regions.

### 2.3. The RFD-BiSeNet V2 Model

**Figure 3:**
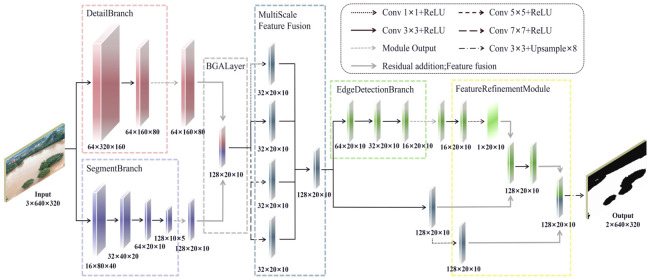
Architecture diagram of the proposed RFD-BiSeNet V2 network.

To address the limitations of BiSeNet V2 in flood segmentation, namely edge blurring, poor multi-scale adaptability, and weak anti-interference capability, this study proposes an improved model named RFD-BiSeNet V2. The model enhances performance through three key innovations: First, a parallel Edge Branch is incorporated, which dynamically integrates edge information into the segmentation process through feature interaction with the main branches. Second, a FRM based on an attention mechanism is introduced to enhance water boundary features and suppress background interference. Finally, a multi-scale feature fusion module is designed to adaptively fuse features from different levels, thereby improving the perception of multi-scale edges. By optimizing the initial weight allocation and learning rate strategy, the model accelerates convergence while ensuring training stability. Experiments demonstrate that RFD-BiSeNet V2 achieves superior boundary precision and robustness in flood segmentation. The overall architecture of the proposed RFD-BiSeNet V2 is depicted in Figure 3.

Given the blurred boundaries between flood and non-flood areas and the issue of class imbalance, RFD-BiSeNet V2 employs a compound loss function for computation, which proves more effective for boundary-sensitive tasks than using cross-entropy loss alone. The compound loss is calculated:

(3) $$L=\alpha \cdot L_{CE}+\beta \cdot L_{Dice},$$

where α=0.6, β=0.4, $L_{CE}$ represents the Cross-Entropy loss.

The Dice Loss, used to measure the overlap between the predicted and ground truth regions, is calculated:

(4) $$L_{Dice}=1-\frac{2\sum_{i}(p_{i}y_{i})+\epsilon }{\sum_{i}p_{i}+\sum_{i}y_{i}+\epsilon },$$

where ϵ is a smoothing factor added to prevent division by zero, typically a small value, e.g., ${1\times 10}^{-6}$.

### 2.4. Specific Improvement Modules

#### 2.4.1. Edge-Aware Learning Strategy

**Figure 4:**
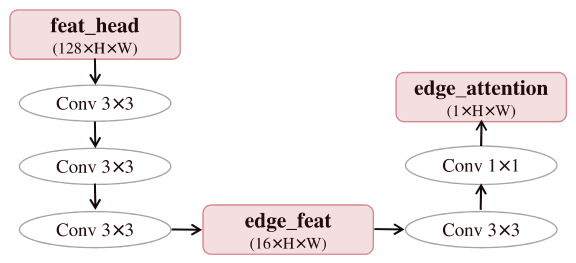
Schematic diagram of the edge-aware learning strategy.

To mitigate the loss of boundary details in BiSeNet V2 for flood segmentation, this study designs a dedicated Edge Branch. The original model suffers from detail loss due to downsampling, and its Bilateral Guidance Aggregation (BGA) module combined with the standard loss function struggles to effectively learn edge pixels. The newly added branch directly locates flood contours by outputting an edge probability map and utilizes these edge features to guide the optimization of the backbone features, thereby enhancing the model’s perception of boundary regions. Concurrently, by introducing an auxiliary boundary learning task, it alleviates the learning bias caused by relying solely on category labels, enabling the model to learn various features more evenly and significantly improving boundary segmentation accuracy. Edge Branch is shown in Figure 4.

To balance edge learning capability with model lightweightness, the Edge Branch is constructed using 3×3 convolutional kernels and shares feature inputs with the backbone network. This branch takes the explicitly output edge probability map as a supervision signal. It is optimized via a dedicated edge loss function to refine the edge structure, thereby guiding the backbone network to increase its sensitivity to boundary regions.

#### 2.4.2. Feature Refinement Module

**Figure 5:**
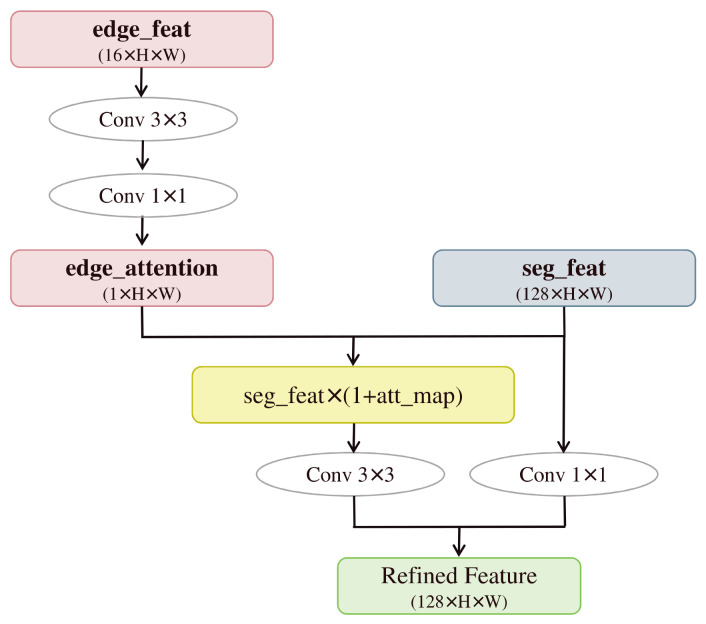
Schematic diagram of the feature refinement module.

Boundary regions are prone to being neglected during training due to their sparse pixels and weak gradients. Furthermore, the backbone network’s inherent emphasis on high-level semantics often leads to the blurring of edge information. Although the original BGA module performs feature fusion, it lacks a fine-grained control mechanism to effectively guide the precise localization of edges. To address this, this study proposes a FRM illustrated in Figure 5, which employs an edge attention mechanism to enhance the responsiveness of semantic features to boundaries. Additionally, by incorporating residual connections, it fuses edge details while maintaining the stability of semantic information, thereby generating more accurate and natural segmentation results.

The FRM operates by fusing semantic and edge features. It generates an edge attention map through convolutional dimensionality reduction and concatenation. This map is then used to modulate the semantic features, enhancing their response at boundary regions. The modulated result undergoes a fusion convolution and is then combined with the original features via a residual connection, ultimately outputting the refined features. This design dynamically optimizes semantic features using edge information, effectively improving boundary segmentation accuracy and the model’s capability to delineate transition areas.

#### 2.4.3. Multi-Scale Feature Fusion Module

**Figure 6:**
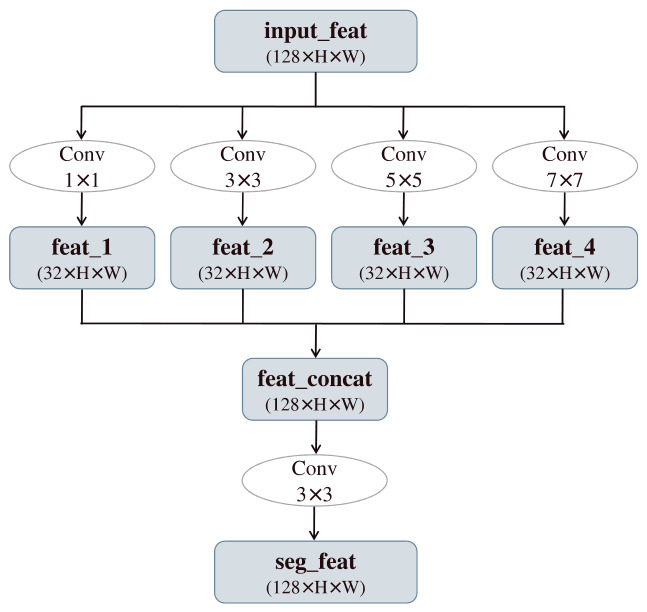
Schematic diagram of the multi-scale feature fusion module.

The original BiSeNet V2 employs only local convolutions after BGA fusion, resulting in a limited receptive field. This makes it difficult to handle objects with large scale variations in complex scenes, leading to significant segmentation errors in details like water edges. Moreover, the semantic branch lacks a multi-scale parsing mechanism, which can easily cause category confusion. To overcome these limitations, this paper introduces a MFFM is shown in Figure 6. This module processes the BGA output in parallel using four convolutional kernels of different sizes: small-scale kernels preserve edge details, while large-scale kernels expand the receptive field to capture global context. The features from these multiple pathways are then concatenated and integrated through a convolutional layer, effectively enhancing the model’s adaptability to multi-scale objects.

This design achieves effective fusion of multi-level features at a low computational cost. It not only significantly improves the model’s ability to preserve details of meandering water boundaries but also enhances its understanding of large-scale water body structures. Consequently, it effectively resolves the original network’s deficiencies in multi-scale perception and edge continuity.

### 2.5. Ablation Experiment

To quantitatively evaluate the effectiveness of each module in the proposed RFD-BiSeNet V2 architecture, an ablation study was conducted on the flood dataset. Using the original BiSeNet V2 as the baseline, three modules—Edge Branch, FRM, and MFFM were progressively integrated. Experimental results are presented in Table 1.

The baseline model achieved 90.42% mIoU. Introducing the Edge Branch improved boundary feature capture, increasing performance to 93.03% mIoU, demonstrating that explicit edge supervision enhances sensitivity to water boundaries in complex flood scenes.

With the addition of the FRM, which refines semantic features and suppresses background interference via an attention mechanism, performance further improved to 94.14% mIoU. The complete model incorporating the MFFM achieved the best results—97.10% mIoU —confirming that multi-scale feature fusion effectively captures water boundaries across spatial scales and ensures segmentation consistency in complex environments.

Regarding computational efficiency, integrating the three modules slightly reduced inference speed from 38.42 FPS to 30.51 FPS, while parameters increased from 5.12 M to 5.95 M.

Nonetheless, the model retains real-time performance and lightweight characteristics. These results indicate that the proposed improvements significantly enhance segmentation accuracy while maintaining a favorable balance between efficiency and model complexity.

## 3. Results

### 3.1. Limitations of the Baseline BiSeNet V2

During the training of the original BiSeNet V2 model, the water segmentation mIoU on the validation set reached 90.42%, indicating its competent performance in segmenting conventional regions. However, its performance significantly declined in challenging flood edge scenarios with complex interference such as water surface reflections, ripples, and strong light glares: edge extraction exhibited irregular fluctuations, violating the inherently continuous and smooth nature of water bodies. Misjudgments created “hollow regions” within contiguous water areas, compromising regional consistency. Concurrently, false detections and missed detections frequently occurred in regions with visually similar features. These phenomena highlight the model’s inadequate adaptability in complex edge scenarios.

### 3.2. Visualization Results of RFD-BiSeNet V2

**Figure 7:**
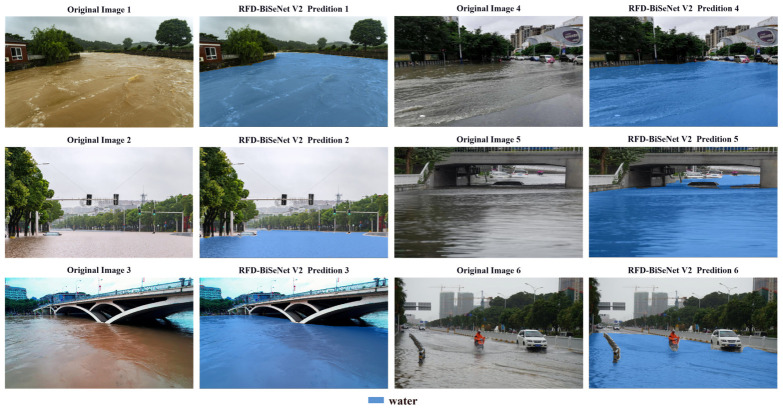
Flood prediction results of RFD-BiSeNet V2.

The proposed RFD-BiSeNet V2 model aims to optimize flood segmentation performance, with prediction results showcased in Figure 7. The blue masks represent the flood areas predicted by the model.

**Figure 8:**
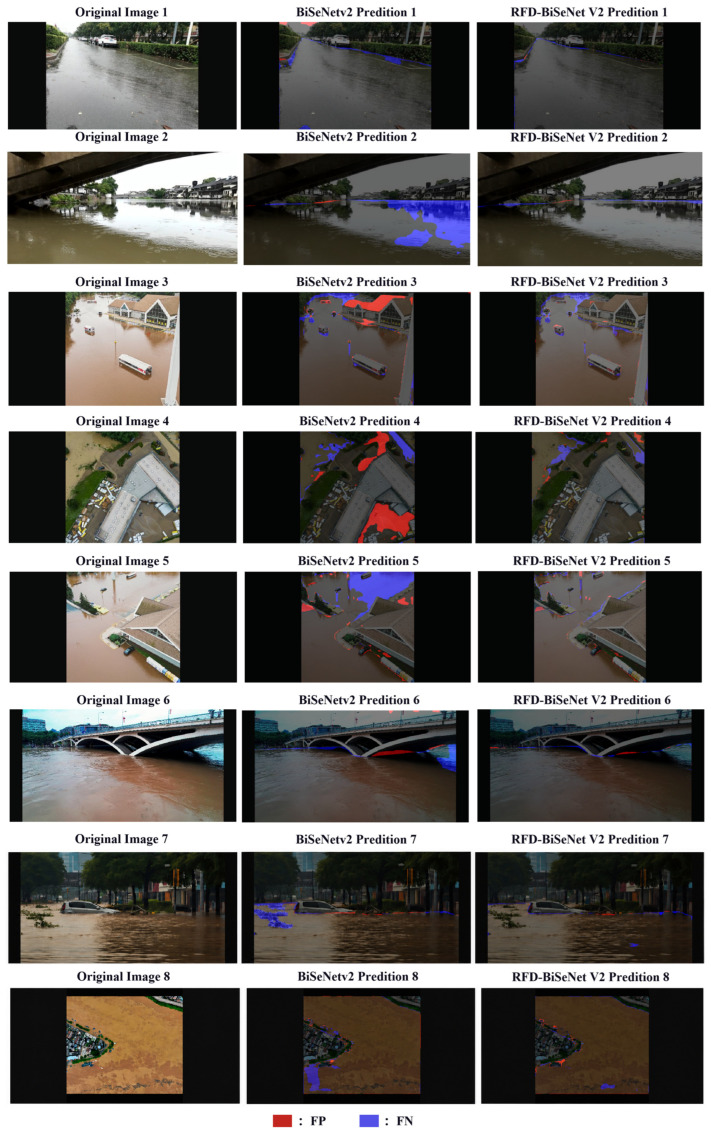
Comparison of prediction results between RFD-BiSeNet V2 and BiSeNet V2.

To visually validate the efficacy of the proposed enhancements, Figure 8 compares the segmentation results of the original BiSeNet V2 and RFD-BiSeNet V2. In these visualizations, red masks denote false positives (areas incorrectly detected as floodwater), while blue masks represent false negatives (missed water areas). Quantitatively, RFD-BiSeNet V2 achieves a substantial 6.68% increase in mIoU over the baseline model. Qualitatively, the predicted water regions exhibit a high degree of congruence with the ground truth. This superiority is particularly pronounced in complex boundary segmentation tasks, where RFD-BiSeNet V2 consistently yields smoother contours and tighter alignment with actual water edges. While minor morphological inconsistencies persist in localized areas featuring extreme turbulence or fragmented debris—an inherent limitation of pixel-level classification devoid of explicit geometric constraints—these localized artifacts do not compromise the macroscopic accuracy of flood extent estimation. Ultimately, the robust and precise floodwater detection capabilities demonstrated by RFD-BiSeNet V2 provide reliable algorithmic support for the deployment of unmanned equipment in time-critical flood rescue operations.

### 3.3. Boundary Segmentation Performance Analysis

The RFD-BiSeNet V2 model enhances its perception of boundary features by introducing a water edge weighting mechanism—assigning higher weights to edge regions during the feature learning phase, which prompts the model to prioritize learning discriminative patterns for boundaries. This improvement directly yields three key optimizations: it enhances the accuracy of water region segmentation, effectively avoiding the false detection of “hollow regions” within water bodies. It improves the smoothness of the predicted edges, making them better conform to the morphological characteristics of real water bodies, and it further improves the identification capability for floodwaters, reducing instances of missed and false detections.

### 3.4. Failure Case Analysis and Limitations

**Figure 9:**
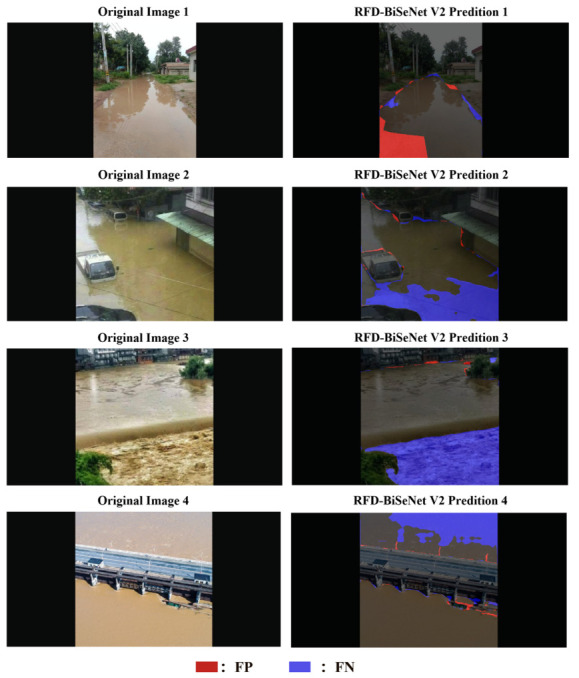
Visualization of typical failure cases involving complex water morphologies.

Despite the high segmentation accuracy of RFD-BiSeNet V2 across diverse scenarios, certain localized omissions persist under extremely complex water surface morphologies. As illustrated in Figure 9, these failure cases primarily occur in turbulent floodwaters characterized by fine water splashes or dense, high-frequency ripples. These dynamic features introduce significant visual noise and irregular reflections, which can disrupt the local semantic consistency and lead the network to misclassify these fragmented regions as background. Nevertheless, such artifacts are typically confined to minute, localized areas and do not compromise the model’s overall efficacy in macroscopic flood extent identification.

## 4. Discussion

### 4.1. Effectiveness of the Proposed Improvement Strategies

The improvement strategies employed in RFD-BiSeNet V2 demonstrate clear effectiveness. The Edge Branch enhances boundary feature learning through explicit edge supervision. The FRM enables the synergistic optimization of semantic and edge features. The multi-scale feature fusion module improves adaptability to complex scenes. and the initial weight allocation strategy accelerates model training convergence and module coordination. These improvements map directly to the observed accuracy gains: the Edge Branch explicitly resolves blurred water edges, the FRM mitigates weak anti-interference capability against reflections, and the MFFM handles large scale variations. Together, they validate the feasibility.

**Figure 10:**
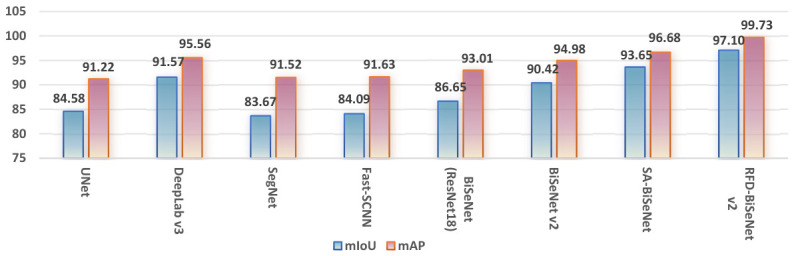
Visual comparison of training results among RFD-BiSeNet V2 and other models.

To comprehensively demonstrate the segmentation performance of different models on the dataset, a multi-metric comparative analysis was conducted, with the results summarized in Figure 10 and Table 2. Furthermore, the specific performance metrics of the proposed model are detailed in Table 3.

### 4.2. Real-Time Performance and Model Efficiency Analysis

Regarding the balance between real-time performance and lightweight design, RFD-BiSeNet V2 maintains an inference frame rate of 30 FPS on GTX 1060 hardware. Although this is lower than BiSeNet V2’s 38 FPS, it still meets real-time segmentation requirements. With a parameter count of 5.95 million, it is reduced by 2.82 million compared to similar optimized models and is substantially lower than traditional models like SegNet, conforming to lightweight design standards. Compared to Fast-SCNN, the RFD-BiSeNet V2 model achieves a 14% increase in segmentation accuracy at the cost of a moderate speed reduction, striking an optimized balance between precision and efficiency.

**Figure 11:**
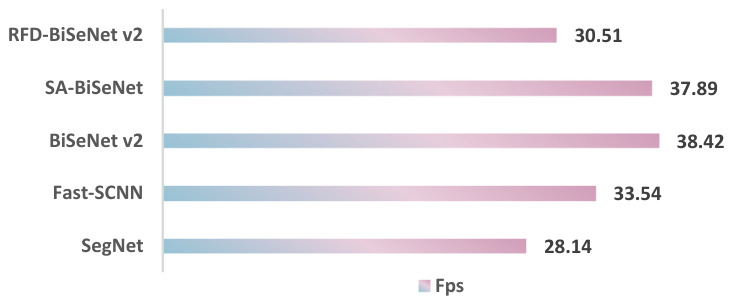
FPS comparison between RFD-BiSeNet V2 and other real-time models.

To comprehensively demonstrate the real-time performance of different models on this dataset, a multi-metric comparative analysis was conducted, with the results summarized in Figure 11 and Table 4.

### 4.3. Cross-Dataset Generalization Analysis

To further evaluate the generalization capability of RFD-BiSeNet V2, zero-shot inference tests were conducted on the publicly available AIFloodSense dataset without any fine-tuning or retraining [31]. The experimental results are summarized in Table 5.

The quantitative data indicates that the model maintains a high precision of 93.16% in a completely unseen testing environment. This demonstrates that the captured flood visual features possess strong universality and can effectively mitigate complex background interference in cross-domain scenarios. However, the recall (74.86%) exhibits a decline compared to the in-domain evaluation. Error analysis reveals that the number of False Negative (FN) samples is approximately five times that of False Positives (FP). This suggests that RFD-BiSeNet V2 exhibits a distinct “conservative” characteristic when processing out-of-distribution data: the model tends to label only water regions with high confidence, ensuring an extremely low false-alarm rate while resulting in certain omissions in highly complex environments.

**Figure 12:**
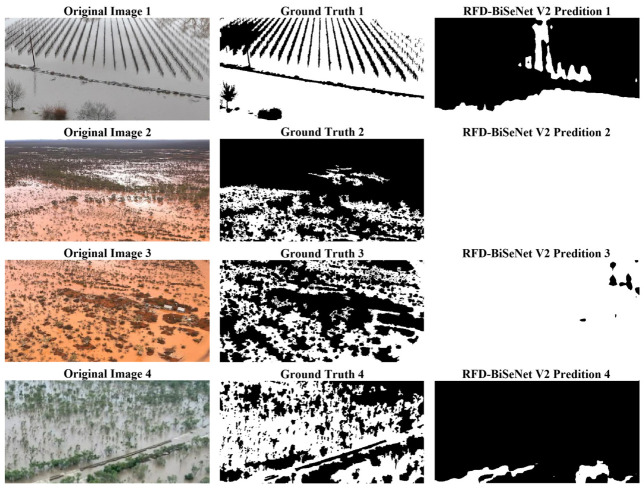
Visualization of typical failure cases on the AIFloodSense dataset.

Further visual analysis identifies typical scenarios where the model performance is constrained. As illustrated in Figure 12, omissions (FNs) are predominantly concentrated in small-scale water bodies or narrow flow boundaries. Since the AIFloodSense dataset contains a large number of high-altitude UAV images where flood areas occupy minimal pixel space, lightweight networks are prone to losing fine-grained spatial details during the deep feature propagation and downsampling processes.

To address the issue of suppressed recall in cross-domain testing, future optimizations can be pursued in three dimensions:

1. **Inference Logic Adjustment**: By appropriately lowering the confidence threshold for water detection during post-processing, the recall can be significantly improved with minimal sacrifice to precision.
2. **Loss Function Reformulation**: Increasing the class weight for water bodies during the training phase to compel the network to prioritize sparse and fragmented water features.
3. **Targeted Data Augmentation**: Supplementing the training set with a higher proportion of “high water-occupancy” and “micro-scale water” samples to enhance the model’s structural robustness across multi-scale flood morphologies.

## 5. Conclusions

Flood disasters pose significant threats to human life and infrastructure, creating an urgent need for reliable vision-based environmental sensing technologies for rapid floodwater identification. To address these challenges across diverse monitoring platforms, including both USVs and UAVs, this study proposes RFD-BiSeNet V2, a lightweight semantic segmentation network designed to improve the balance between segmentation accuracy and real-time performance. Based on the BiSeNet V2 framework, the proposed model introduces an edge-aware learning strategy, a feature refinement module (FRM), and a multi-scale feature fusion module to enhance boundary perception and suppress background interference in complex flood environments. Evaluated on a diverse dataset incorporating USV data and UAV imagery, the proposed method achieves an mIoU of 97.10%, outperforming the baseline by 6.68%. The model successfully maintains lightweight characteristics with 5.95M parameters and achieves real-time inference at 30.51 FPS, proving its viability for resource-constrained deployments.

Furthermore, zero-shot cross-dataset evaluation demonstrates the model’s strong generalization capability and high precision in completely unseen environments. While it exhibits robust anti-interference performance, evaluation on high-altitude UAV datasets indicates potential for improved recall regarding micro-scale water bodies and narrow flow boundaries. Consequently, future work will focus on optimizing cross-domain performance through inference logic adjustments, loss function reformulation, and targeted data augmentation for small-scale water features. Additionally, to maximize its practical impact in emergency response, future efforts will explore deploying the model on embedded edge devices via quantization and pruning, as well as integrating the extracted 2D masks with 3D geospatial data to develop immersive AR/VR flood visualizations [32,33].

## Figures and Tables

**Table 1:** Ablation experiment of the proposed modules on the flood dataset.

Model	Edge-Aware Learning Strategy	Feature Refinement Module	Multi-Scale Feature Fusion Module	mIoU (%)	FPS	Params (M)
BiSeNet V2 (baseline)	×	×	×	90.42	38.42	5.12
+Edge Branch	√	×	×	93.03	35.84	5.41
+Edge Branch + FRM	√	√	×	94.14	32.76	5.68
+Edge Branch + FRM+ MFFM	√	√	√	97.10	30.51	5.95

**Table 2:** Performance Comparison of RFD-BiSeNet V2 with Other Models.

Model	mIoU %
UNet [11]	84.58
DeepLab v3 [10]	91.87
SegNet [28]	83.67
Fast-SCNN [29]	84.09
BiSeNet (ResNet18) [25]	86.65
BiSeNet V2 [22]	90.42
SA-BiSeNet [30]	93.65
RFD-BiSeNet V2	97.10

**Table 3:** Performance evaluation of RFD-BiSeNet V2 on the flood dataset.

Model	mIoU %	Precision %	Recall %	$F_{1}$-Score %
RFD-BiSeNet V2	97.10	98.87	96.94	98.06

**Table 4:** FPS comparison of RFD-BiSeNet V2 and other real-time models on GTX 1060.

Model	Fps
SegNet [11]	28.14
Fast-SCNN [29]	33.54
BiSeNet V2 [22]	38.42
SA-BiSeNet [30]	37.89
RFD-BiSeNet V2	30.51

**Table 5:** Zero-shot inference results of the proposed model on the AIFloodSense dataset.

Metric	Value %	Metric	Value %
Precision	93.17	Water IoU	70.98
Recall	74.87	mIoU	76.25
$F_{1}$-score	83.02	Pixel Acc	87.28

## Data Availability

The original contributions presented in this study are included in the article. Further inquiries can be directed to the corresponding author.
